# Can we use Ki67 expression to predict prostate cancer aggressiveness?

**DOI:** 10.1590/0100-6991e-20223200-en

**Published:** 2022-06-23

**Authors:** RONALDO MAIA, GABRIEL ARANTES DOS SANTOS, SABRINA REIS, NAYARA I VIANA, RUAN PIMENTA, VANESSA R GUIMARÃES, SAULO RECUERO, POLIANA ROMÃO, KATIA RAMOS MOREIRA LEITE, MIGUEL SROUGI, CARLO CARMARGO PASSEROTTI

**Affiliations:** 1 - Hospital Alemão Oswaldo Cruz, Center for Robotic Surgery - São Paulo - SP - Brasil; 2 - Faculdade de Medicina da Universidade de São Paulo (FMUSP), Urologia - São Paulo - SP - Brasil; 3 - D’Or Institute for Research and Education (IDOR) - São Paulo - SP - Brasil; 4 - Hospital Moriah - São Paulo - SP - Brasil; 5 - Universidade do Estado de Minas Gerais (UEMG) - Passos - MG - Brasil

**Keywords:** Biomarkers, Tumor, Prognosis, Immunoassay, Biomarcadores Tumorais, Imuno-Histoquímica, Prognóstico

## Abstract

**Introduction:**

specialists have an urge for biomarkers that can discriminate indolent prostate cancer from aggressive tumors. Ki67 is a proliferation marker, and its expression is associated with the aggressiveness of several cancers.

**Objective:**

analyze the expression of Ki67 in prostate cancer samples correlating with the aggressiveness of the disease.

**Methods:**

Ki67 mRNA levels were determined utilizing data from a TCGA cohort (Tumor(n)=492 and control(n)=52). The protein expression was determined on 94 biopsies from patients by immunohistochemical assay.

**Results:**

in mRNA, the Ki67 upregulation is associated with cancer tissue (p<0.0001) and worst disease-free survival (p=0.035). The protein upregulation is associated with increase of the ISUP score (p<0.0001), cancer stage (p=0.05), biochemical recurrence (p=0.0006) and metastasis (p<0.0001). We also show a positive correlation between Ki67 expression and ISUP score (r=0.5112, p<0.0001) and disease risk stratification (r=0.3388, p=0.0009). Ki67 expression is a factor independently associated with biochemical recurrence (p=0.002) and metastasis (p<0.0001). Finally, the patients with high Ki67expression shows better survival regarding biochemical recurrence (p=0.008) and metastasis (p=0.056). Patients with high Ki67 expression are 2.62 times more likely to develop biochemical recurrence (p=0.036).

**Conclusion:**

Ki67 upregulation is associated with prostate cancer aggressiveness.

## RESUMO

**Introdução:**

especialistas precisam biomarcadores que podem discriminar o câncer de próstata indolente de tumores agressivos. Ki67 é um marcador de proliferação, e sua expressão está associada à agressividade de vários tumores.

**Objetivo:**

analisar a expressão do Ki67 em amostras de câncer de próstata correlacionando com a agressividade da doença.

**Métodos:**

os níveis de mRNA de Ki67 foram determinados utilizando dados de uma coorte de TCGA (Tumor(n)=492 e controle(n)=52). A expressão da proteína foi determinada em 94 biópsias de pacientes por ensaio imuno-histoquímica.

**Resultados:**

no mRNA, a superexpressão Ki67 está associada ao tecido canceroso (p<0,0001) e à pior sobrevida livre de doença (p=0,035). A superexpressão proteica está associada ao aumento do escore ISUP (p<0,0001), estágio de câncer (p=0,05), recorrência bioquímica (p=0,0006) e metástase (p<0,0001). Também mostramos uma correlação positiva entre a expressão Ki67 e o escore ISUP (r=0,5112, p<0,0001) e a estratificação de risco de doença (r=0,3388, p=0,0009). A expressão Ki67 é um fator independentemente associado à recorrência bioquímica (p=0,002) e metástase (p<0,0001). Finalmente, os pacientes com alta expressão de Ki67 expression mostram melhor sobrevivência em relação à recorrência bioquímica (p=0,008) e metástase (p=0,056). Os pacientes com alta expressão de Ki67 são 2,62 vezes mais propensos a desenvolver recorrência bioquímica (p=0,036).

**Conclusão:**

a superexpressão Ki67 está associada à agressividade do câncer de próstata.

## INTRODUCTION

Prostate cancer (PC) is one of the most prevalent cancers globally and the cause of death of thousands of men every year^1^. Although radical prostatectomy (the most common treatment for PC) has good results in general, it has some side effects, such as erectile dysfunction and urinary incontinence^2^.

Since overtreatment is a challenge in PC, it is critical to predict which patients need invasive treatment since they harbor aggressive cancer and which would have indolent disease. Due to the significant heterogeneity of prostate cancer, classical prognostic factors, such as serum PSA, pathological staging, and Gleason score, are not sufficiently accurate to separate indolent from aggressive cancers in a reliable way^3^. In this sense, molecular prognostic biomarkers can be essential tools in the clinical management of PC.

The Ki67 protein, widely used as a proliferation marker, is expressed in all cell cycle phases, except G0 and G1^4^. Because of this, the potential as a prognostic biomarker of this protein is evaluated in several types of cancer, such as breast, lung, bladder, gastric, and prostate cancer^5^ ^-^ ^9^. In addition, the use of Ki67 is potentially applicable, as it is technically accessible and easy to interpret^10^.

Whereas there is a need for new molecular biomarkers to classify PC, in this manuscript, we evaluated whether the expression of Ki67 can predict the aggressiveness of the disease.

## METHODS

### Ethics

This study was submitted and approved by the Research Ethics Committee of the Medical School of the University of São Paulo under number 3,407,345. All participants signed the informed consent form and were informed about safety in terms of integrity.

### TCGA cohort

We use RNA-seq data from The Cancer Genome Atlas (TCGA) PC datasets. The cohort consisted of 492 samples of prostate adenocarcinoma and 52 samples of paired normal samples.

All analyses were made using the online Gene Expression Profiling Interactive Analysis (GEPIA) database^11^. The unit of gene expression is Transcripts per Million. All images are original from GEPIA, with minor styles adjustments.

### Patients

A total of 94 biopsies from patients with prostate cancer, treated surgically in 1998, 1999, 2006, and 2007, at Hospital das Clínicas of the Medical School of University of São Paulo, were selected. To obtain clinical and etiological data, electronic medical records were evaluated. The characteristics of the patients are summarized in Table 1.

**Table 1:** Patients Characteristics.

Age (years)	62.88 (±6.95)
PSA (ng/ml)	10.19 (±13.12)
ISUP (n)	
1	23
2	16
3	20
4	23
5	12
Stage (n)	
pT2	50
pT3	44
Age (years)	62.88 (±6.95)
Biochemical Recurrence (n)	
-	58
+	25
no information	11
Metastasis (n)	
-	64
+	12
no information	18
Risk Stratification (n)	
Very low	0
Low	15
Favorable intermediary	8
Unfavorable intermediary	11
High	60

### Immunohistochemical assay

Protein expression was assessed by immunohistochemistry with the construction of a Tissue Microarray. 

The immunohistochemical assay was performed on five slides containing the PC specimens simultaneously, allowing homogeneity in the evaluations. First, the deparaffinization of the Tissue Microarray slides was carried out in an oven at 60-65ºC for 1 hour and washed with Xylol and decreasing dilutions of alcohol followed by washing with distilled water. Then, the antigen was recovered by heat in a universal Diva antigen recovery buffer (1:100) and heated for 10 minutes at 110ºC in an electric pressure cooker (Decloacker).

Next, slides were cooled at room temperature for 20 minutes and incubated overnight at 4ºC with the Ki67 monoclonal antibody (Orb7758, Biobyt). The LSAB system was used for immunostaining (LSAB; Dako Cytomation, CA, USA). Staining was carried out using a 3,3’-diaminobenzidine-chromogen substrate solution, followed by counterstaining with Harris’ hematoxylin. The slides were then dehydrated, mounted with coverslips, and observed in an optical microscope by an expert pathologist.

### Analysis of results

The graphs and the statistical analysis were performed using GraphPad Prism 8 SPSS software (23.0). We used Student’s t-test for two groups and one-way ANOVA with Bonferroni’s correction for three or more groups for the hypothesis test. 

Correlation analysis used Pearson’s r test for the hypothesis test. Logistic regression is applied to evaluate if Ki67 expression is independently associated with prognostic factors. For all statistical analyses, we set a level of significance of 5% (p≤0.05).

## RESULTS

### Ki67 mRNA upregulation is associated with the onset and progression of Prostate Cancer

**Figure 1:**
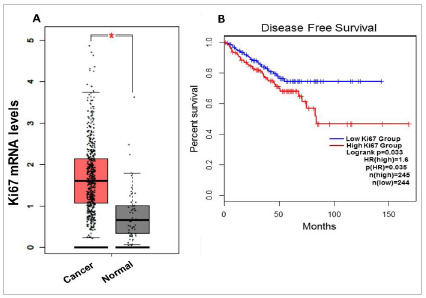
Ki67 mRNA levels in prostate cancer. In A Ki67, transcription is upregulated in cancer (Log2Fold Change Cutoff=0.9). In B, the upregulation of Ki67 is associated with worse disease-free survival. *p<0.01; HR= Hazard Ratio.

To analyze the transcription of Ki67 in prostate cancer, we utilized TCGA PC datasets. When we compared the expression of Ki67 mRNA between cancer and normal tissue, we observed a significant upregulation (p<0.01) of the transcript in the disease (Figure 1A).

Next, we separated the tumors according to mRNA expression and observed patients with the worst disease-free survival overexpressed Ki67 (HR=1.6, p=0.035) (Figure 1B).

### Upregulation of Ki67 protein expression is associated with prostate cancer aggressiveness

**Figure 2:**
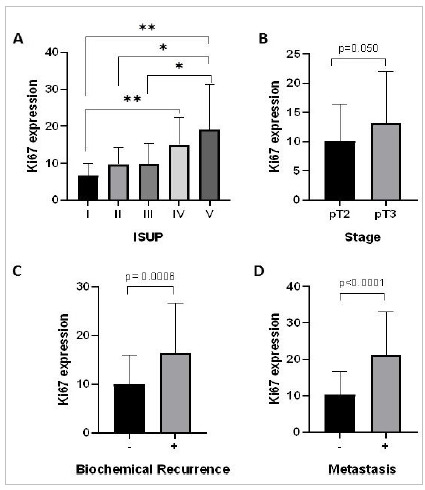
Association between Ki67 protein expression and prostate cancer aggressiveness. In A, we have the progressive Ki67 upregulation with the increase of the ISUP score. In B, we have the upregulation of Ki67 associated with the extraprostatic disease. In C and D, we have the upregulation of Ki67 associated with the presence of biochemical recurrence and metastasis, respectively. **p<0.001, *p<0.01.

Then, by immunohistochemical assay, we checked whether Ki67 protein levels were associated with the aggressiveness of primary PC in clinical samples of the disease. The progressive upregulation of Ki67 is associated with the increase of the International Society of Urological Pathology score (ISUP) (p<0.0001) (Figure 2A). In particular, ISUP 4 and ISUP 5 tumors shows the highest expression, with significant differences between ISUP 1 and ISUP 4 (p=0.0009) and between ISUP 5 and ISUP 1, 2 and 3 (p<0.0001, p=0.0046 and p= 0.0027, respectively). 

The upregulation of Ki67 was also associated with the non-confined disease (Figure 2B) and the presence of biochemical recurrence (Figure 2C) and metastasis (Figure 2D) (p=0.05, p=0.0006, and p<0.0001, respectively). No association between PSA levels and Ki67 expression was found (data not showed).

### Quantitative analysis showed that the upregulation of Ki67 correlates with a worse prognosis for prostate cancer

For a possible application of Ki67 expression in the clinical practice of PC, we need to evaluate its expression quantitatively. For this, we categorize our patients taking into account the ISUP score and the risk stratification for prostate cancer (AUA/ASTRO/SUO Guideline^3^ ^,^ ^12^), and performed a correlation analysis. The increase in Ki67 correlates positively with the worst prognosis of the disease, considering both the ISUP score (r=0.5112, p<0.0001) and risk stratification (r=0.3388, p=0.0009) (Table 2).

**Table 2:** Correlation between Ki67 expression and prognostic factors.

Pearson r	ISUP	Risk Stratification
R	0.5112	0.3388
95% confidence interval	0.3433 to 0.6475	0.1451 to 0.5075
p value	<0.0001	0.0009

In addition, we performed a logistic regression to identify whether Ki67 expression is independently associated with prognostic factors. In this result, we found that Ki67 expression was a factor independently associated with biochemical recurrence (p=0.002) and metastasis (p<0.0001).

Finally, we categorize the expression of Ki67 to its median. Then, we performed Kaplan-Meier survival curves and Cox analysis to analyze whether values above the median are related to biochemical recurrence and metastasis. Patients with lower Ki67 expression had a better survival curve considering biochemical recurrence (Figure 3A, p=0.0008). Concerning metastasis, the high expression of Ki67 was associated with the worst survival curve but with a marginal p-value, which indicates a trend (Figure 3B, p=0.056).

Next, we showed that PSA and Ki67 expression categorization could predict biochemical recurrence (Table 3, p=0.006 and p=0.036, respectively). None of the variables analyzed can predict, with statistical significance, metastasis.

**Table 3:** Cox Analysis.

Biochemical Recurrence			
	Hazard Ratio	p value	95.0% CI
PSA	3.475	0.006	1.442 ~ 8.378
Ki67	2.62	0.036	1.066 ~ 6.438
Stage	1.469	0.413	0.585 ~ 3.693
ISUP	1.032	0.845	0.751 ~ 1.418
Metastasis			
	Hazard Ratio	p value	95.0% CI
PSA	3.238	0.063	0.936 ~ 11.2
Ki67	2.966	0.178	0.61 ~14.412
Stage	2.632	0.194	0.612 ~ 11.327
ISUP	1.121	0.629	0.704 ~1.785

## DISCUSSION

Usually, early detection of cancer is associated with better results, but for patients with PC, this can also result in overdiagnosis and overtreatment, stressing the need for new biomarkers that could predict the aggressiveness of the disease^13^. In this sense, using molecular markers can improve personalized medicine, improving the clinical management of patients with PC^14^. In this work, we analyzed whether the expression of Ki67, a classic cancer proliferation marker, can be used to predict PC aggressiveness.

A recent study reevaluates the use of classical prognostic factors of PC (Gleason score, PSA, etc.) for the staging of neoplasia, and despite demonstrating important results, a limitation that the authors discuss is the non-use of molecular biomarkers^15^. The most recent ASCO guideline for molecular biomarkers in localized prostate cancer states that, although the expression of Ki67 can offer information on the diagnosis/prognosis of the disease, there is currently insufficient evidence to support its clinical use^16^. 

Considering mRNA levels, we analyzed TCGA datasets and showed that the upregulation of Ki67 is associated with the malignant tissue and the worst disease-free survival. This suggests that Ki67 has a role in the onsets of carcinogenesis and the progression of cancer.

Although TCGA has already provided critical data on the biogenesis of PC, in the routine, immunohistochemical assays are more accessible because they show the protein expression of the biomarker and are already used by most pathologists^17^. Using this technique, we found that the upregulation of Ki67 is associated with a higher ISUP score, extraprostatic disease, biochemical recurrence, and metastasis. Curiously, we did not find any association between PSA levels and Ki67 expression, and some authors discuss that although PSA is important for disease screening, the prognostic value of PSA levels is low and have been associated with a high rate of overdiagnosis/overtreatment in clinical trials^18^ ^-^ ^20^. The use of Ki67 expression can help to discriminate when increased PSA is clinically significant.

In a more quantitative approach, we demonstrate a positive correlation between Ki67 expression and ISUP score and disease risk stratification. This is important because both classifications are recent and consider disease-specific survival curves^3^ ^,^ ^21^. There is a consensus among urologists that biochemical recurrence is associated with a poor prognosis and metastasis with PC worst (and potentially fatal) state. Here we show that positive regulation of Ki67 is an independent predictor of these two factors^22^. Patients with high Ki67 expression show the worst survival curves regarding biochemical recurrence and metastasis. Besides that, in our cohort, the patients with high Ki67 expression are 2.62 times more likely to develop biochemical recurrence, suggesting that this protein may be a predictor of more aggressive cancers.

Ki67 expression shows no association with PC prognosis in a relatively old study^23^. Despite this, more recent studies generally agree that upregulation of Ki67 is associated with the worsening of the disease. A paper showed that Ki67 expression predicts biochemical recurrence and death from PC^24^. Additionally, a multicenter study confirms that Ki67 is an independent predictor of biochemical recurrence after radical prostatectomy^25^. Finally, an article with more than 1000 surgical specimens of PC concludes that high Ki67 expression was strongly associated with a higher Gleason score, cancer stage, seminal vesicle invasion, extracapsular extension, and the greater probability of disease recurrence^26^.

In summary, by mRNA and protein levels, we showed that the upregulation is associated with the aggressiveness of PC. Our results corroborate similar observations in the literature, and with that, we propose that the Ki67 immunohistochemical assay should be incorporated into the prognostic evaluation of PC^9^ ^,^ ^27^ ^-^ ^30^.

## References

[1] Siegel RL, Miller KD, Jemal A (2020). Cancer statistics, 2020. CA Cancer J Clin.

[2] Mottet N BJ, Briers E, Bolla M, Cornford P, De Santis M (2020). European Association of Urology Prostate Cancer Guidelines.

[3] Sanda MG, Cadeddu JA, Kirkby E, Chen RC, Crispino T, Fontanarosa J (2018). Clinically Localized Prostate Cancer AUA/ASTRO/SUO Guideline. Part I: Risk Stratification, Shared Decision Making, and Care Options. J Urol.

[4] Cher ML, Chew K, Rosenau W, Carroll PR (1995). Cellular proliferation in prostatic adenocarcinoma as assessed by bromodeoxyuridine uptake and Ki-67 and PCNA expression. Prostate.

[5] Yerushalmi R, Woods R, Ravdin PM, Hayes MM, Gelmon KA (2010). Ki67 in breast cancer prognostic and predictive potential. Lancet Oncol.

[6] Grant L, Banerji S, Murphy L, Dawe DE, Harlos C, Myal Y (2018). Androgen Receptor and Ki67 Expression and Survival Outcomes in Non-small Cell Lung Cancer. Horm Cancer.

[7] He Y, Wang N, Zhou X, Wang J, Ding Z, Chen X (2018). Prognostic value of ki67 in BCG-treated non-muscle invasive bladder cancer a meta-analysis and systematic review. BMJ Open.

[8] Böger C, Behrens HM, Röcken C (2016). Ki67--An unsuitable marker of gastric cancer prognosis unmasks intratumoral heterogeneity. J Surg Oncol.

[9] Lobo J, Rodrigues Â, Antunes L, Graça I, Ramalho-Carvalho J, Vieira FQ (2018). High immunoexpression of Ki67, EZH2, and SMYD3 in diagnostic prostate biopsies independently predicts outcome in patients with prostate cancer. Urol Oncol.

[10] Hayes DF, Allen J, Compton C, Gustavsen G, Leonard DG, McCormack R (2013). Breaking a vicious cycle. Sci Transl Med.

[11] Tang Z, Li C, Kang B, Gao G, Zhang Z (2017). GEPIA a web server for cancer and normal gene expression profiling and interactive analyses. Nucleic Acids Res.

[12] Sanda MG, Cadeddu JA, Kirkby E, Chen RC, Crispino T, Fontanarosa J (2018). Clinically Localized Prostate Cancer AUA/ASTRO/SUO Guideline. Part II: Recommended Approaches and Details of Specific Care Options. J Urol.

[13] Miyamoto DT, Lee RJ, Stott SL, Ting DT, Wittner BS, Ulman M (2012). Androgen receptor signaling in circulating tumor cells as a marker of hormonally responsive prostate cancer. Cancer Discov.

[14] Di Sanzo M, Cipolloni L, Borro M, La Russa R, Santurro A, Scopetti M (2017). Clinical Applications of Personalized Medicine A New Paradigm and Challenge. Curr Pharm Biotechnol.

[15] Dess RT, Suresh K, Zelefsky MJ, Freedland SJ, Mahal BA, Cooperberg MR (2020). Development and Validation of a Clinical Prognostic Stage Group System for Nonmetastatic Prostate Cancer Using Disease-Specific Mortality Results From the International Staging Collaboration for Cancer of the Prostate. JAMA Oncol.

[16] Eggener SE, Rumble RB, Armstrong AJ, Morgan TM, Crispino T, Cornford P (2020). Molecular Biomarkers in Localized Prostate Cancer ASCO Guideline. J Clin Oncol.

[17] Kristiansen G (2018). Markers of clinical utility in the differential diagnosis and prognosis of prostate cancer. Mod Pathol.

[18] Hugosson J, Carlsson S, Aus G, Bergdahl S, Khatami A, Lodding P (2010). Mortality results from the Göteborg randomised population-based prostate-cancer screening trial. Lancet Oncol.

[19] Andriole GL, Crawford ED, Grubb RL, Buys SS, Chia D, Church TR (2009). Mortality results from a randomized prostate-cancer screening trial. N Engl J Med.

[20] Schröder FH, Hugosson J, Roobol MJ, Tammela TL, Ciatto S, Nelen V (2009). Screening and prostate-cancer mortality in a randomized European study. N Engl J Med.

[21] Epstein JI, Egevad L, Amin MB, Delahunt B, Srigley JR, Humphrey PA (2016). The 2014 International Society of Urological Pathology (ISUP) Consensus Conference on Gleason Grading of Prostatic Carcinoma Definition of Grading Patterns and Proposal for a New Grading System. Am J Surg Pathol.

[22] Artibani W, Porcaro AB, De Marco V, Cerruto MA, Siracusano S (2018). Management of Biochemical Recurrence after Primary Curative Treatment for Prostate Cancer A Review. Urol Int.

[23] Nariculam J, Freeman A, Bott S, Munson P, Cable N, Brookman-Amissah N (2009). Utility of tissue microarrays for profiling prognostic biomarkers in clinically localized prostate cancer the expression of BCL-2, E-cadherin, Ki-67 and p53 as predictors of biochemical failure after radical prostatectomy with nested control for clinical and pathological risk factors. Asian J Androl.

[24] Desmeules P, Hovington H, Nguilé-Makao M, Léger C, Caron A, Lacombe L (2015). Comparison of digital image analysis and visual scoring of KI-67 in prostate cancer prognosis after prostatectomy. Diagn Pathol.

[25] Mathieu R, Shariat SF, Seitz C, Karakiewicz PI, Fajkovic H, Sun M (2015). Multi-institutional validation of the prognostic value of Ki-67 labeling index in patients treated with radical prostatectomy. World J Urol.

[26] Tretiakova MS, Wei W, Boyer HD, Newcomb LF, Hawley S, Auman H (2016). Prognostic value of Ki67 in localized prostate carcinoma a multi-institutional study of &gt;1000 prostatectomies. Prostate Cancer Prostatic Dis.

[27] Byun SS, Lee M, Hong SK, Lee H (2019). Elevated Ki-67 (MIB-1) expression as an independent predictor for unfavorable pathologic outcomes and biochemical recurrence after radical prostatectomy in patients with localized prostate cancer A propensity score matched study. PLoS One.

[28] Fantony JJ, Howard LE, Csizmadi I, Armstrong AJ, Lark AL, Galet C (2018). Is Ki67 prognostic for aggressive prostate cancer A multicenter real-world study. Biomark Med.

[29] Wilkins AC, Gusterson B, Szijgyarto Z, Haviland J, Griffin C, Stuttle C (2018). Ki67 Is an Independent Predictor of Recurrence in the Largest Randomized Trial of 3 Radiation Fractionation Schedules in Localized Prostate Cancer. Int J Radiat Oncol Biol Phys.

[30] Lindsay CR, Le Moulec S, Billiot F, Loriot Y, Ngo-Camus M, Vielh P (2016). Vimentin and Ki67 expression in circulating tumour cells derived from castrate-resistant prostate cancer. BMC Cancer.

